# Supplementary material for: Identification of evolutionarily conserved virulence factor by selective pressure analysis of Streptococcus pneumoniae
Source: Commun Biol. 2019 Mar 8;2:96. doi: 10.1038/s42003-019-0340-7 (PMC6408437; doi:10.1038/s42003-019-0340-7)
Supplement: Supplementary file 1 — Reporting Summary [file 42003_2019_340_MOESM1_ESM.pdf]

nature research

Corresponding author(s): Masaya Yamaguchi

Last updated by author(s): Jan 23, 2019

Reporting Summary

Nature Research wishes to improve the reproducibility of the work that we publish. This form provides structure for consistency and transparency in reporting. For further information on Nature Research policies, see Authors & Referees and the Editorial Policy Checklist.

Statistics

For all statistical analyses, confirm that the following items are present in the figure legend, table legend, main text, or Methods section.

n/a	Confirmed	
☐	☒	The exact sample size (n) for each experimental group/condition, given as a discrete number and unit of measurement
☐	☒	A statement on whether measurements were taken from distinct samples or whether the same sample was measured repeatedly
☐	☒	The statistical test(s) used AND whether they are one- or two-sided<br>*Only common tests should be described solely by name; describe more complex techniques in the Methods section.*
☒	☐	A description of all covariates tested
☐	☒	A description of any assumptions or corrections, such as tests of normality and adjustment for multiple comparisons
☐	☒	A full description of the statistical parameters including central tendency (e.g. means) or other basic estimates (e.g. regression coefficient) AND variation (e.g. standard deviation) or associated estimates of uncertainty (e.g. confidence intervals)
☐	☒	For null hypothesis testing, the test statistic (e.g. F, t, r) with confidence intervals, effect sizes, degrees of freedom and P value noted<br>*Give P values as exact values whenever suitable.*
☐	☒	For Bayesian analysis, information on the choice of priors and Markov chain Monte Carlo settings
☒	☐	For hierarchical and complex designs, identification of the appropriate level for tests and full reporting of outcomes
☒	☐	Estimates of effect sizes (e.g. Cohen's d, Pearson's r), indicating how they were calculated

Our web collection on statistics for biologists contains articles on many of the points above.

Software and code

Policy information about availability of computer code

Data collection	tBLASTn function of NCBI BLAST
Data analysis	FGENESB (Bacterial Operon and Gene Prediction) and BPROM: http://www.softberry.com/berry.phtml?topic=index&group=programs&subgroup=gfindb<br>Phylogears2 and Kakusan4: https://www.fifthdimension.jp/<br>MAFFT v.7.221: https://mafft.cbrc.jp/alignment/software/<br>Jalview v.2.9.0b2: http://www.jalview.org/<br>MrBayes v.3.2.5: http://mrbayes.sourceforge.net/<br>RAxML v.8.1.20: https://sco.h-its.org/exelixis/software.html<br>FigTree v.1.4.2: http://tree.bio.ed.ac.uk/software/figtree/<br>HyPhy software v.2.220150316beta(MP) for MacOS(Universal binary): http://www.hyphy.org/<br>Prism v6.0h & v.7.0d software (Graphpad software)<br>MOTIF search: https://www.genome.jp/tools/motif/<br>DMFit program: https://www.combase.cc

For manuscripts utilizing custom algorithms or software that are central to the research but not yet described in published literature, software must be made available to editors/reviewers. We strongly encourage code deposition in a community repository (e.g. GitHub). See the Nature Research guidelines for submitting code & software for further information.

Data

Policy information about availability of data

All manuscripts must include a data availability statement. This statement should provide the following information, where applicable:

- Accession codes, unique identifiers, or web links for publicly available datasets
- A list of figures that have associated raw data
- A description of any restrictions on data availability

There are no restrictions on data availability. Accession codes are provided in this paper.

Field-specific reporting

Please select the one below that is the best fit for your research. If you are not sure, read the appropriate sections before making your selection.

☒ Life sciences ☐ Behavioural & social sciences ☐ Ecological, evolutionary & environmental sciences

For a reference copy of the document with all sections, see nature.com/documents/nr-reporting-summary-flat.pdf

Life sciences study design

All studies must disclose on these points even when the disclosure is negative.

Sample size	Sample size was determined following preliminary experiments in order to limit mice number in agreement with the rules of the local ethic committee on animal experimentations. Experimental size numbers were based on previous experiments (Yamaguchi M. et al. Sci. Rep. 2016, Henningham A. et al. J. Biol. Chem. 2014, Mori Y. et al. J. Biol. Chem. 2012, Yamaguchi M. et al. J. Biol. Chem. 2008).
Data exclusions	In quantitative PCR, one well was omitted. It is because the well was shown as an outlier by the PCR system, STEPOne program.
Replication	Data from human primary cells, cell lines, and in vivo experiments were generated using at least three independent biological replicates and we showed the data in this manuscript from a representative experiment. Quantitative PCR data were pooled and normalised from three or four independent experiments, each performed in triplicate.
Randomization	For in vivo experiments, mice were randomized matching for age, weight, and sex.
Blinding	No blinding was performed in these experiments.

Reporting for specific materials, systems and methods

We require information from authors about some types of materials, experimental systems and methods used in many studies. Here, indicate whether each material, system or method listed is relevant to your study. If you are not sure if a list item applies to your research, read the appropriate section before selecting a response.

Materials & experimental systems

n/a	Involved in the study	
☒	☐	Antibodies
☐	☒	Eukaryotic cell lines
☒	☐	Palaeontology
☐	☒	Animals and other organisms
☐	☒	Human research participants
☒	☐	Clinical data

Methods

n/a	Involved in the study	
☒	☐	ChIP-seq
☒	☐	Flow cytometry
☒	☐	MRI-based neuroimaging

Eukaryotic cell lines

Policy information about cell lines

Cell line source(s)	A549 (ATCC CCL-185) was obtained from RIKEN Cell Bank (Japan).
Authentication	Identity of the cells was frequently checked by their morphological features but has not been authenticated by the short tandem repeat (STR) profiling.
Mycoplasma contamination	All cell lines were tested negative for mycoplasma contamination.
Commonly misidentified lines (See ICLAC register)	No commonly misidentified cell lines were used.

Animals and other organisms

Policy information about studies involving animals; ARRIVE guidelines recommended for reporting animal research

Laboratory animals	Mouse, females, CD1, 6 weeks old
Wild animals	This study did not involve wild animals.
Field-collected samples	This study did not involve samples collected from the wild.
Ethics oversight	All mouse experiments were conducted in accordance with animal protocols approved by the Animal Care and Use Committee of Osaka University Graduate School of Dentistry (28-002-0).

Note that full information on the approval of the study protocol must also be provided in the manuscript.

Human research participants

Policy information about studies involving human research participants

Population characteristics	This study involves the use of human blood and neutrophils isolated from peripheral blood from healthy volunteers. Blood was collected via venopuncture from healthy donors after obtaining written, informed consent according to a protocol approved by the institutional review board of Osaka University Graduate School of Dentistry (H26-E43).
Recruitment	Healthy donors were recruited in Osaka University Graduate School of Dentistry.
Ethics oversight	As described above, the protocol approved by the institutional review board of Osaka University Graduate School of Dentistry (H26-E43).

Note that full information on the approval of the study protocol must also be provided in the manuscript.
