# Supplementary material for: Identification of evolutionarily conserved virulence factor by selective pressure analysis of Streptococcus pneumoniae
Source: Commun Biol. 2019 Mar 8;2:96. doi: 10.1038/s42003-019-0340-7 (PMC6408437; doi:10.1038/s42003-019-0340-7)
Supplement: Supplementary file 3 — Description of Additional Supplementary Files [file 42003_2019_340_MOESM3_ESM.docx]

**Description of Additional Supplementary Files**

**Supplementary Data 1**

Locus tag number of *cbp* genes in each pneumococcal strain. The data is source for phylogenetic and evolutional analyses (Fig. 2, Fig. 3, Fig. 4, and Table 1).

**Supplementary Data 2**

Figure 5 and 6 source data. The tabs in this excel file correspond to panels of each figure, as labelled.
